# High incidence of Hepatitis C virus infection observed in the PROUD study of HIV pre‐exposure prophylaxis

**DOI:** 10.1111/jvh.13297

**Published:** 2020-04-13

**Authors:** Monica Desai, Ellen White, Nina Vora, Richard Gilson, Charles Lacey, Mitzy Gafos, Amanda Clarke, Ann Sullivan, David White, Julie Fox, David Piontkowsky, Sheena McCormack, David T. Dunn

**Affiliations:** ^1^ MRC Clinical Trials Unit at UCL London UK; ^2^ UCL Centre for Clinical Research in Infection and Sexual Health Institute for Global Health The Mortimer Market Centre London UK; ^3^ Hull York Medical School University of York York UK; ^4^ Brighton and Hove Sexual Health & Contraception Centre Royal Sussex County Hospital Brighton UK; ^5^ Chelsea and Westminster NHS Foundation Trust London UK; ^6^ Heart of England NHS Foundation Trust Birmingham UK; ^7^ Guy’s and St Thomas’ NHS Foundation Trust London UK; ^8^ Gilead Sciences Foster City CA USA; ^9^ Present address: National Institute for Health and Care Excellence Manchester UK; ^10^ Present address: London School of Hygiene and Tropical Medicine London UK

**Keywords:** hepatitis C, incidence, pre‐exposure prophylaxis

**Abbreviations**
MSM: sex with men
PrEP: pre‐exposure prophylaxis
HCV: hepatitis C virus infection
PY: person‐years

## 1 INTRODUCTION

In general, HIV‐negative men who have sex with men (MSM) have been considered to be at low risk for hepatitis C virus (HCV) infection.^1^, ^2^ HIV‐negative MSM who access pre‐exposure prophylaxis (PrEP) have reported sexual behaviours that could place them at high risk of HCV, including high partner numbers, chemsex and injecting drug use.^3^, ^4^ Early diagnosis of HCV infection allows early linkage to treatment and care, and reduction in onward transmission of infection.^5^ Data from European PrEP trials and cohort studies of PrEP users have reported high baseline HCV prevalence and incidence during follow‐up.^6^, ^7^, ^8^ In contrast, studies from North America have generally found low levels of HCV endemicity among PrEP users.^9^, ^10^, ^11^

The PROUD study was an open‐label trial of HIV PrEP among 544 HIV‐negative MSM.^12^, ^13^ As there were no data on HCV incidence in HIV‐negative MSM using PrEP in the UK, we implemented routine quarterly screening during the long‐term follow‐up phase when all participants had access to PrEP. We report HCV prevalence and incidence among participants in the PROUD study.

## 2 MATERIALS AND METHODS

PROUD was an open‐label, wait‐list trial design that randomized MSM attending participating sexual health centres in England to receive HIV PrEP immediately or after a deferral period of 1 year (the deferred phase). Five hundred and forty‐four participants were recruited between November 2012 and April 2014, and follow‐up continued to October 2016. The protocol was modified in November 2014 following an interim analysis which showed PrEP to be highly effective, resulting in some participants in the deferred arm being offered PrEP earlier than one year (N = 163).^13^

The initial PROUD protocol followed national guidelines on HCV testing, with screening 'on indication'. Screening at enrolment was not mandated. The tests used during the trial varied by site, and the use of antibody, antigen or viral load tests depending on whether the participant had a prior history of HCV. Information on HCV was collected in a number of ways. At enrolment, participants self‐reported a diagnosis with HCV in the previous 12 months, and the clinician reported whether the participant had ever been screened for, and if so, diagnosed with HCV. At each visit, the number of HCV screens and positive screens (although not distinguishing the type of test) since the previous visit was recorded. For all new infections, clinics were asked to provide detailed clinical and laboratory information, including dates of last positive and last negative test (for all assays), HCV viral load test results, liver function test results and history of injecting drug use. In March 2015, additional funding was acquired which allowed screening at every quarterly study visit; at this time, all participants had access to PrEP and the trial was closed to further recruitment.

If the first HCV antibody (anti‐HCV) test was negative, the participant was assumed to be seronegative at enrolment. If the first test was positive, the Trial Management Group determined whether infection was most likely acquired before enrolment (and thus contributed to the seroprevalence analysis) or after enrolment (and thus contributed to the incidence analysis) based on alanine transaminase and HCV viral load measurements, in relation to time since randomization.

### 2.1 Statistical analysis

The cumulative incidence of HCV infection (time to diagnosis) was estimated using Kaplan‐Meier analysis and randomized groups compared with the log‐rank test, censoring at the time of the last screen for HCV. Estimation of incidence by calendar year was complicated by the highly variable time between the last negative test and the first positive test which, in some cases, could span adjacent calendar years. To address this, the date of infection was imputed 1000 times assuming a uniform distribution, and calculating the incidence for each calendar year within each imputed dataset.^14^ Estimates were obtained by averaging across the imputed datasets, and confidence intervals derived using Rubin's rule.^15^ All analyses were done in STATA version 15.1.

## 3 RESULTS

**Figure 1:**
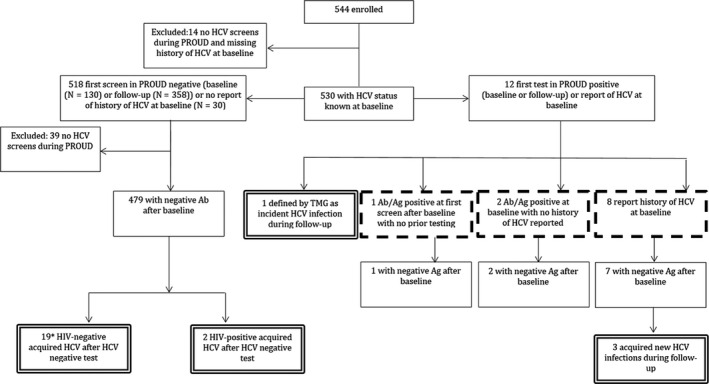
Hepatitis C screening and infection consort diagram. Seroprevalent infections indicated by dashed box. Incidence infections indicated by double‐lined box. TMG, Trial Management Group

Characteristics of the 544 study participants have been previously described.^13^ Figure 1 illustrates the hepatitis C screening and infection among study participants. One hundred and thirty‐three (24.4%) participants were screened for HCV at enrolment, and 499 (91.7%) were tested at least once during follow‐up. Nine participants were only screened at baseline and therefore could not contribute to an incidence analysis. A HCV screen was conducted at 54.0% (3213/5946) of visits (higher during the phase of routine quarterly screening [80.6%] compared to the earlier phase of testing on indication [34.9%]), with a median of 6 (IQR: 3‐8) screens per participant. Of the 45 participants who were never tested, 14 participants were also missing information on HCV history collected at enrolment.

### 3.1 Hepatitis C seroprevalence at enrolment

The seroprevalence at enrolment was 2.1% (11/530; 95% CI: 1.0%‐3.7%). The 11 cases were identified as follows: eight participants were reported by the clinician at enrolment to have had a previous diagnosis of HCV; two were diagnosed with HCV a few days before enrolment; two participants had HCV viraemia detected at their first post‐enrolment test (they were not tested at enrolment), of which one was judged to have acquired infection before enrolment.

### 3.2 Hepatitis C incidence

HCV incidence is based on the 490 participants who were considered HCV seronegative at enrolment, or who had previously cleared HCV infection prior to enrolment, and had at least one post‐enrolment HCV test. Table 1 presents baseline characteristics of these participants and indicates a cohort with high‐risk behaviours: 226 (47.6%) reported use of chemsex‐associated drugs in the past three months, 282 (57.6%) were diagnosed with any STI in the past 12 months, 170 (36.9%) reported using post‐exposure prophylaxis in the past 12 months, and the median number of partners in the past three months was 10.

**Table 1:** Baseline characteristics of PROUD participants included in HCV incidence analysis

Characteristic	N (%) (Total = 490)
Age (median)	35 (29‐42)
Ethnicity	
White	395 (81.4%)
Black	17 (3.5%)
Asian	28 (5.8%)
Other	45 (9.3%)
University degree	303 (62.3%)
Full‐time employment	351 (72.4%)
Born in the UK	292 (60.1%)
Relationship status	
Partner, living together	145 (29.8%)
Partner, living separately	74 (15.2%)
Single	267 (54.9%)
Circumcised	142 (29.4%)
Chemsex‐associated drugs*	226 (47.6%)
STI diagnosed in past 12 mo	
Any**	282 (57.6%)
Rectal***	162 (34.8%)
Rectal*** or syphilis	183 (39.3%)
Number of sexual partners in past 3 mo, median	10 (5‐20)
Number of ncRAI partners in past 3 mo, median	2 (1‐5)
Number of HIV tests in past 12 mo	3 (2‐4)
Use of PEP in past 12 mo	170 (36.9%)

Note

Data are median (IQR) or n (%). Number of participants missing baseline data: ethnicity, 5; university degree, 4; full‐time employment, 5; relationship status, 4; chemsex, 15; STI, 24; total number of partners, 8; ncRAI partners, 25; HIV test, 17; PEP, 29.

Abbreviations: HCV, hepatitis C virus; HIV, human immunodeficiency virus; ncRAI, receptive anal intercourse without a condom; PEP, post‐exposure prophylaxis; STI, sexually transmitted infection; UK, United Kingdom.

* Methamphetamine, GHB, mephedrone or ketamine.

** Chlamydia, gonorrhoea, or syphilis.

*** Rectal chlamydia or rectal gonorrhoea.

The median follow‐up (enrolment to last HCV test) was 2.6 (IQR: 2.1‐3.0) years, with a total follow‐up of 1188.8 person‐years (PY). Overall, 25 participants had a new HCV infection, yielding an incidence rate of 2.1 per 100 PY (25/1188.8; 95% CI: 1.4‐3.1). Three of these were re‐infections (the previous infections had cleared spontaneously or with treatment before enrolment), and one participant experienced two infections during follow‐up (only the first infection was included in the incidence calculation). Two HCV infections were acquired after diagnosis of HIV whilst the participant was no longer on PrEP but was being actively followed up. Excluding these cases from the HCV incidence calculation reduced the estimate only slightly (1.9 per 100 PY [95% CI: 1.2‐2.9]). Use of nonprescribed injected drugs was reported by 11 participants at the suspected time of HCV infection, was denied by 12 and was unknown for two.

**Figure 2:**
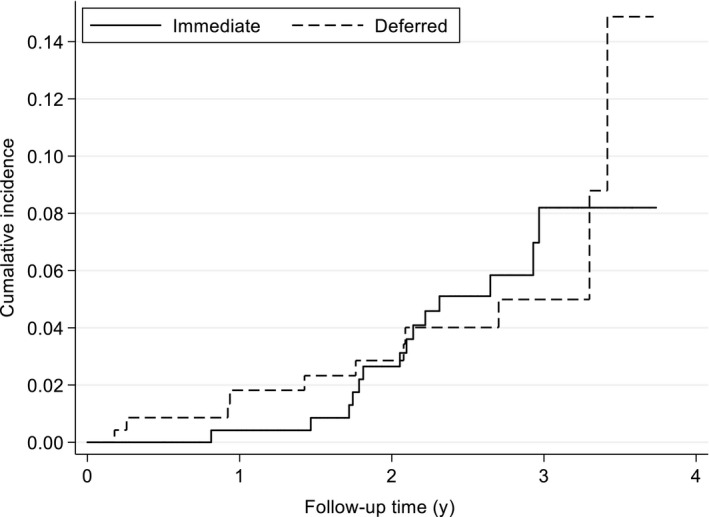
Cumulative incidence of HCV infection (time from enrolment to diagnosis). For illustrative purposes, one participant (IMM) diagnosed 4 d before the end of the trial is excluded. (*P*‐value for log‐rank test = 0.87)

Figure 2 shows the cumulative incidence of time to a new HCV diagnosis, stratified by randomized arm (*P*‐value log‐rank test = 0.87). As only three infections (one immediate, two deferred) were observed during the deferred phase of the study (ie the period before the DEF arm had access to PrEP), the trial essentially provides no randomized information of whether access to PrEP affects the risk of acquiring HCV infection. Accounting for uncertainty in the time of acquisition of HCV infection, HCV incidence appeared to increase over calendar time (Table 2), reaching an estimated 4.0 per 100 PY in 2016 (95% CI: 2.0‐8.1, *P*‐value for trend = 0.09).

**Table 2:** HCV incidence stratified by calendar year

Year	Person‐years follow‐up	Estimated no. of HCV infections^a^	Rate per 100 PY (95% CI)*
2013	154.8	2.8	1.8 (0.5‐6.2)
2014	431.4	5.7	1.3 (0.5‐3.3)
2015	393.2	8.2	2.1 (1.0‐4.4)
2016	205.1	8.2	4.0 (2.0‐8.1)

^a^ Accounting for infection interval over calendar years.

* *P*‐value for trend = 0.09.

## 4 DISCUSSION

This analysis demonstrates a high overall incidence of hepatitis C infection (2.1 per 100 PY) in the PROUD trial. This is higher than, although compatible with, the incidence reported in other contemporaneous PrEP studies in Europe. In the AmPrEP study, HCV incidence during follow‐up was 1.9 per 100 PY (95% CI: 1.1‐3.4). The incidence rate of primary infection was 1.0 per 100 PY (95% CI: 0.5‐2.2) and of re‐infection 25.5 per 100 PY (95% CI: 11.5‐56.8).^6^ In the ANRS IPERGAY study of on‐demand PrEP, HCV incidence was estimated to be 1.4 per 100 PY (95% CI: 0.7‐2.4).^7^ A large French cohort estimated HCV incidence to be 1.2 per 100 PY for HIV‐negative MSM PrEP users.^8^

In contrast, demonstration and implementation cohorts of PrEP users in North America have reported lower incidence of HCV infection. An incidence of 0.7 per 100 PY (95% CI: 0.08‐2.4) was reported from a San Francisco clinic.^10^ A retrospective cohort study in a Montreal sexual health clinic that compared the incidence of STIs prior to, and in 12 months following, the prescription of PrEP in 109 MSM found no incident cases of HCV during either time period.^9^ The disparity in HCV incidence seen between European and North American PrEP studies could be due to a different risk of exposure to the virus (ie variation in the prevalence of active infection among MSM) or differences in the extent of high‐risk sexual behaviours. PROUD participants were at the far end of this spectrum, reporting an average of 10 sexual partners in the three months before enrolment and 37% having used post‐exposure prophylaxis in the previous 12 months (Table 1).

Few studies have estimated HCV incidence in general HIV‐negative MSM populations. A meta‐analysis by Ghisla et al estimated incidence to be 0.43 per 1000 PY (95% CI: 0.01‐0.86).^2^ A cohort in Amsterdam did not observe any HCV infections in HIV‐negative participants during 7808 PY of follow‐up (0 per 1000 PY, 95% CI: 0.0‐0.5).^16^ In a UK (Brighton) hospital, HCV incidence was estimated at 0.15 per 100 PY (95% CI: 0.05‐0.35)^17^ among 57% of eligible participants who were tested. The difference in HCV incidence between HIV‐negative MSM in general and those seeking PrEP is likely to be explained by the lower sexual risk behaviours and lower testing rates in the former group.

Although the increase in HCV incidence over calendar timein PROUD was not conventionally statistically significant, the low seroprevalence at enrolment (2.1%, coincidently identical to the overall incidence of 2.1% per year) suggests that the increasing incidence is genuine. This increase occurred despite an overall decline in HCV prevalence in England in recent years.^18^ Phylogenetic analysis of HCV infections in the AmPrEP study and a cohort of HIV‐negative and HIV‐positive MSM in a French clinic^19^, ^20^ suggest that there was substantial sexual mixing between HIV‐negative and HIV‐positive populations, who have high HCV prevalence^21^ and incidence,^2^, ^22^ and it is likely that PrEP facilitates this mixing. Although the frequency of HCV testing increased part way through the study and probably resulted in more rapid diagnosis of infections, we used an imputation method that should correct for any bias arising from this.

The 2.1% seroprevalence of HCV in PROUD participants at enrolment was considerably lower than that reported in the AmPrEP demonstration project (4.8%).^19^ The IPERGAY study reported only one HCV infection at enrolment.^7^ However, HCV seroprevalence changes rapidly in the context of a high incidence and comparisons between studies will be affected by the calendar time over which seroprevalence was calculated.

Our study has two major limitations. First, the small number of incident HCV infections limited our ability to examine risk factors for the acquisition of HCV, including geographical region. The second limitation is around the generalizability of our findings. PROUD participants were at much higher risk of acquiring HIV infection than other MSM attending sexual health clinics in the same time period, and the same may apply to HCV infection. The use of nonprescribed injected drugs was reported by 11 of the 25 incident cases, and this cannot be excluded as a possible route of transmission. However, qualitative research indicates that sharing of needles is uncommon among MSM injecting chemsex drugs in the UK, with high awareness of the risks of doing so.^23^ Even if these 11 cases are discounted, the estimated incidence is still high, suggesting that HCV is highly transmissible through sexual contact, with the risk of epidemic spread of the infection in certain populations.

Regular care for PrEP provides an opportunity to screen for and provide early intervention for HCV. The increasing availability of HCV antigen/antibody testing makes screening in this population more feasible.^24^ The high incidence of HCV that we observed in PROUD supports the 2018 BHIVA/BASHH recommendation for quarterly HCV testing among HIV‐negative MSM using PrEP in the UK, in line with other STIs.^25^ Also, a recent modelling study has indicated that screening and treating PrEP users for HCV at least every 12 months can reduce HCV incidence by 67.3% (uncertainty range 52.7%‐79.2%).^26^ However, HCV incidence can vary markedly by location and time, and guidance in the UK and other countries^27^, ^28^ should be regularly reviewed in the light of local evidence.

## CONFLICTS OF INTERESTS

The PROUD study was provided drug free of charge by Gilead Sciences plc. that also distributed it to participating clinics and provided funds for additional diagnostic tests for HCV and drug levels. EW university fees and stipend funded by Gilead Science plc. SM reports grants from the European Union H2020 scheme, EDCTP 2, the National Institute of Health Research and Gilead Sciences; other support from Gilead Sciences and the Population Council Microbicide Advisory Board; and is Chair of the Project Advisory Committee for USAID grant awarded to CONRAD to develop tenofovir‐based products for use by women (nonfinancial). DTD has received fees for participation on advisory boards and educational workshops from ViiV Healthcare and Gilead Sciences. AC has received consultancy fees from Gilead and ViiV/GSK, and travel bursaries to education events from Gilead. DP is an employee of Gilead Sciences. NV has received conference support from Gilead and Janssen. MD, DW, AS, MG, JF, CL and RG have no conflicts to declare.
